# Supplementary material for: Multimodal cell-free DNA whole-genome TAPS is sensitive and reveals specific cancer signals
Source: Nat Commun. 2025 Jan 8;16:430. doi: 10.1038/s41467-024-55428-y (PMC11711490; doi:10.1038/s41467-024-55428-y)
Supplement: Supplementary file 1 — Supplementary Information [file 41467_2024_55428_MOESM1_ESM.pdf]

Supplementary Information

Multimodal cell-free DNA whole-genome TAPS is sensitive and reveals specific cancer signals

Dimitrios V. Vavoulis[1,2*], Anthony Cutts[1], Nishita Thota[3], Jordan Brown[3], Robert Sugar[3], Antonio Rueda[3], Arman Ardalan[1], Kieran Howard[1], Flavia Matos Santo[1], Thippesh Sannasiddappa[3], Bronwen Miller[3], Stephen Ash[4], Yibin Liu[5,6], Chun-Xiao Song[4,7], Brian D. Nicholson[8], Helene Dreau[1], Carolyn Tregidgo[3], Anna Schuh[1*]

[1]Oxford Molecular Diagnostic Centre, Department of Oncology, University of Oxford, Oxford, UK
[2]Biomedical Research Centre, Centre for Human Genetics, University of Oxford, Oxford, UK
[3]Exact Sciences Innovation LTD, The Sherard Bldg, Edmund Halley Rd, Littlemore, Oxford OX4 4DQ
[4]Ludwig Institute for Cancer Research, Nuffield Department of Medicine, University of Oxford, Oxford, UK
[5]College of Chemistry and Molecular Sciences, Wuhan University, Wuhan 430072, China
[6]Taikang Centre for Life and Medical Sciences, Wuhan University, Wuhan 430072, China
[7]Target Discovery Institute, Nuffield Department of Medicine, University of Oxford, Oxford, UK
[8]Nuffield Department of Primary Care Health Sciences, University of Oxford, Oxford, UK

*Corresponding authors: dimitris.vavoulis@oncology.ox.ac.uk, anna.schuh@oncology.ox.ac.uk

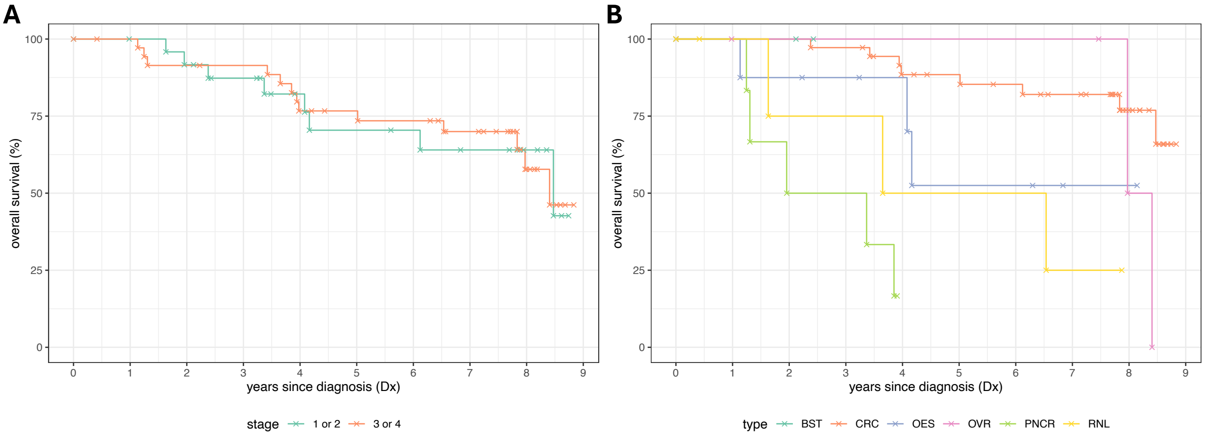

Supplementary Figure 1: *Overall survival by cancer stage and type.* A) Overall survival of cases with confirmed cancer by stage. Median overall survival for both early-stage (1 or 2) and late-stage (3 or 4) disease is approximately 8.5 years since diagnosis. B) Overall survival by cancer type. Patients with colorectal cancer had the longest median overall survival (>8.8 years), followed by oesophageal (>8.1 years), ovarian (8 years), renal (3.7 years), breast (>2.3 years) and pancreatic (2 years) cancer patients. **CRC:** colorectal (n = 36 subjects); **OES:** oesophageal (n = 8 subjects); **PNCR:** pancreatic (n = 6 subjects); **RNL:** renal (n = 5 subjects); **OVR:** ovarian (n = 4 subjects); **BST:** breast (n = 2 subjects). Source data is provided as a source data file.

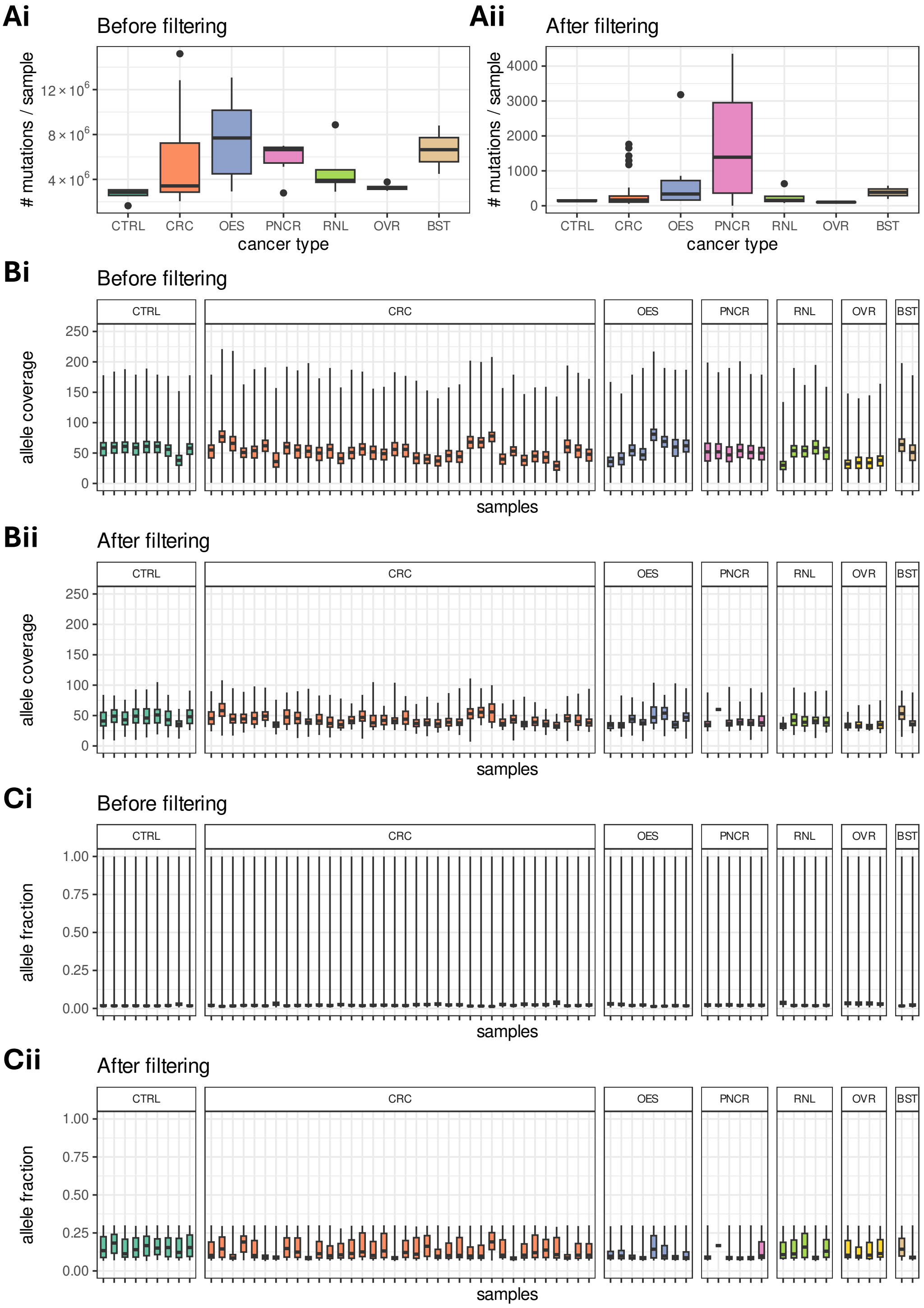

Supplementary Figure 2: ***Filtering somatic single nucleotide variants (SNVs) and indels (INDELs)*****:** Number of SNVs/INDELs, allele coverage and variant allele fraction per sample before (A, C, E) and after (B, D, F) filtering. The impact of filtering was the reduction of the number of SNVs/INDELs per sample (A vs B), as well as removal of the extremes from the SNVs/INDELs coverage (C vs D) and allele fraction (E vs F) distributions in each sample. **CTRL:** CBS controls (n = 9 subjects); **CRC:** colorectal (n = 36 subjects); **OES:** oesophageal (n = 8 subjects); **PNCR:** pancreatic (n = 6 subjects); **RNL:** renal (n = 5 subjects); **OVR:** ovarian (n = 4 subjects); **BST:** breast (n = 2 subjects). Source data is provided as a source data file.

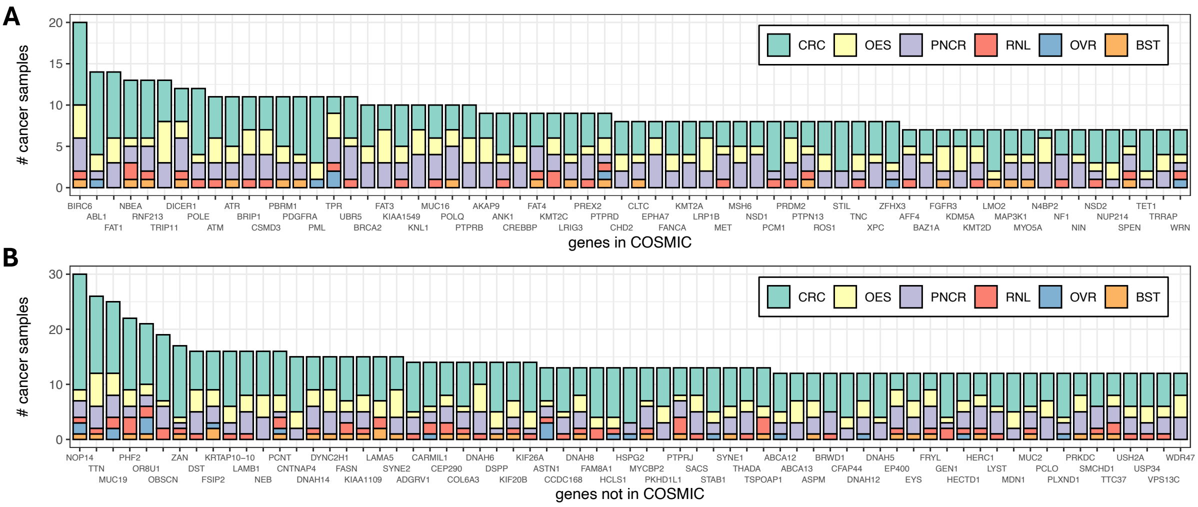

Supplementary Figure 3: *Frequency of mutated genes.* Number of samples mutated in genes found (A) and not found (B) in COSMIC. Only the top 66 genes are shown in each panel, due to space restrictions. **CTRL:** CBS controls (n = 9 subjects); **CRC:** colorectal (n = 36 subjects); **OES:** oesophageal (n = 8 subjects); **PNCR:** pancreatic (n = 6 subjects); **RNL:** renal (n = 5 subjects); **OVR:** ovarian (n = 4 subjects); **BST:** breast (n = 2 subjects). Source data is provided as a source data file.

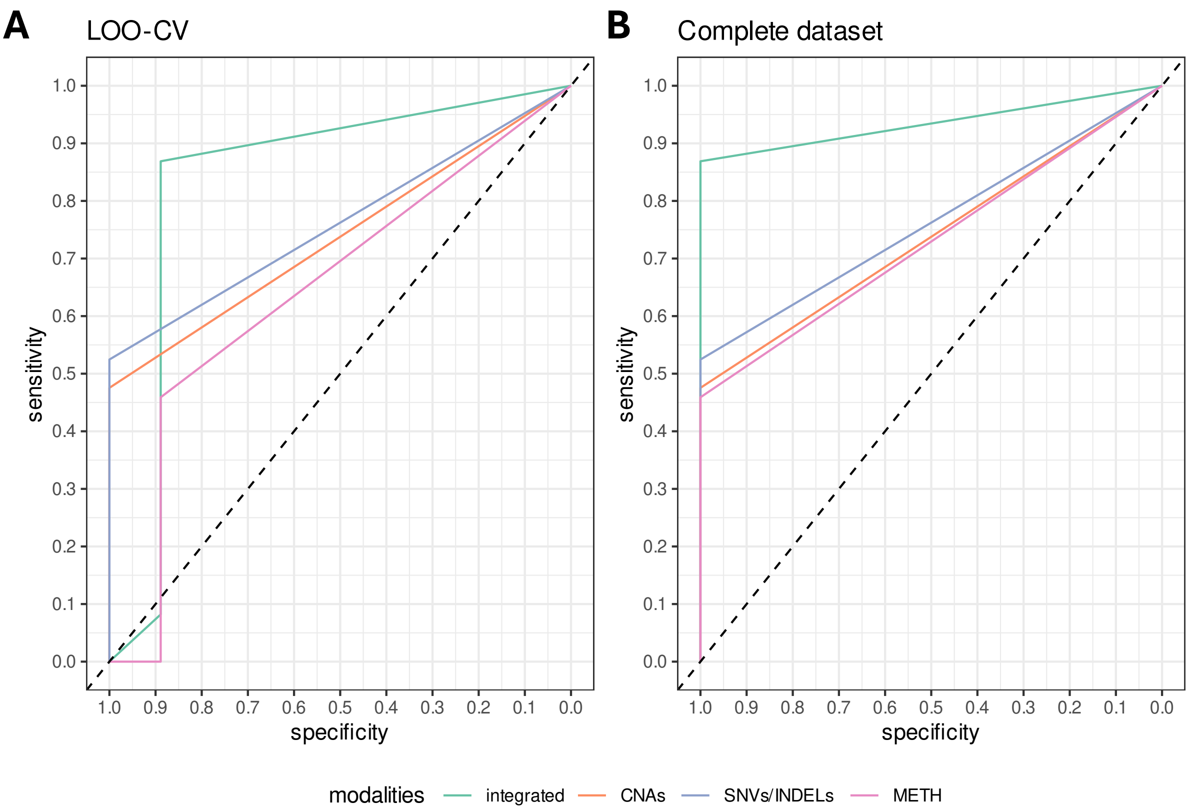

Supplementary Figure 4: Stouffer's method *validation using leave-one-out cross-validation (LOO-CV).* A) We conducted LOO-CV analysis to estimate the out-of-sample prediction error (see *Methods*). The ROC curves of the three data modalities were comparable, but their integration led to a clear boost in predictive performance with 85.2% sensitivity, 88.8% specificity (due to a single false positive) and 83.5% AUC. B) For completeness, we also present the ROC curves for the whole (i.e., non-cross-validated) dataset, where the sensitivity was 85.2%, the specificity was 100%, and the AUC was 93.4%, after integrating all three data modalities. Source data is provided as a source data file.

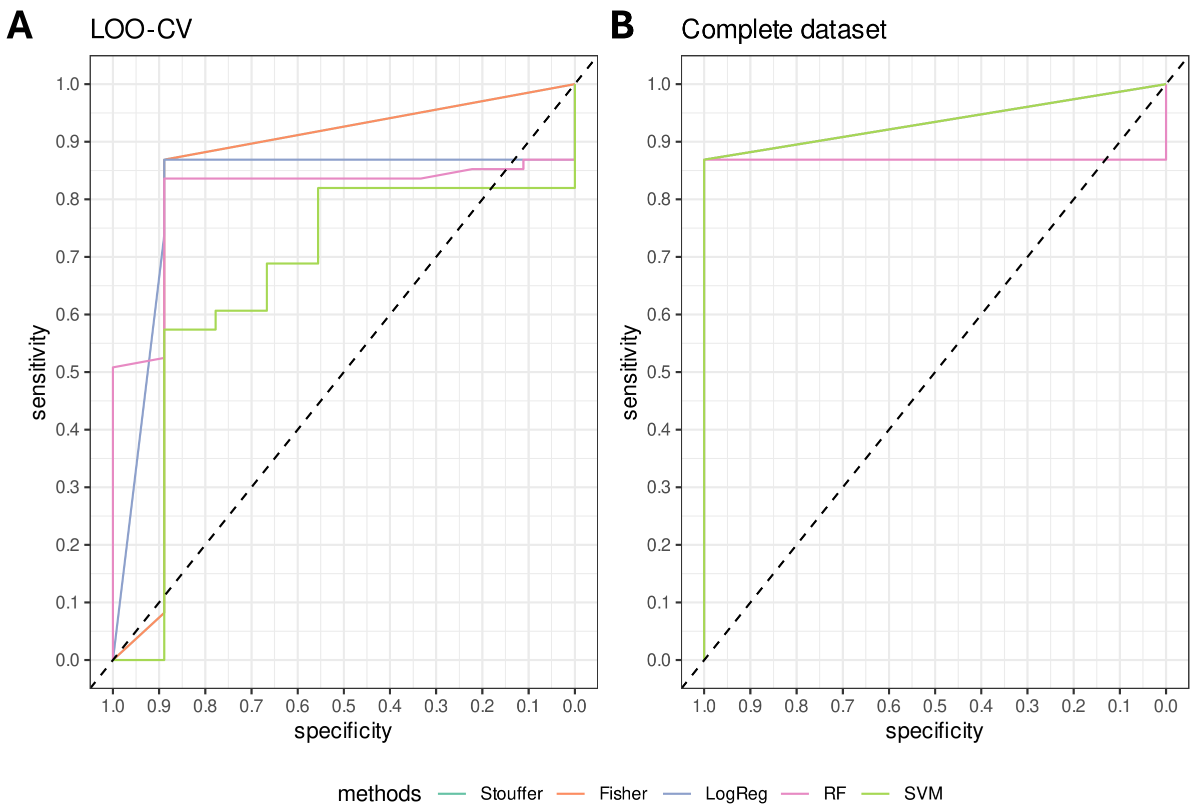

Supplementary Figure 5: *Methods comparison using Leave-One-Out Cross-Validation (LOO-CV).* A) We conducted LOO-CV analysis to estimate the out-of-sample prediction error for a range of methods used for integrating different data modalities (see *Methods*). The best performing methods were Stouffer's and Fisher's (83.5% AUC; ROC curves overlap), followed by Logistic Regression (**LogReg**; 81.3% AUC), Random Forrest (**RF**; 80.7% AUC) and Support Vector Machine (**SVM**; 66.3%). B) For completeness, we also present the corresponding ROC curves for the whole (i.e., non-cross-validated) dataset. All methods perform equally (93.4% AUC; ROC curves overlap), except Random Forrest (86.9% AUC). Source data is provided as a source data file.

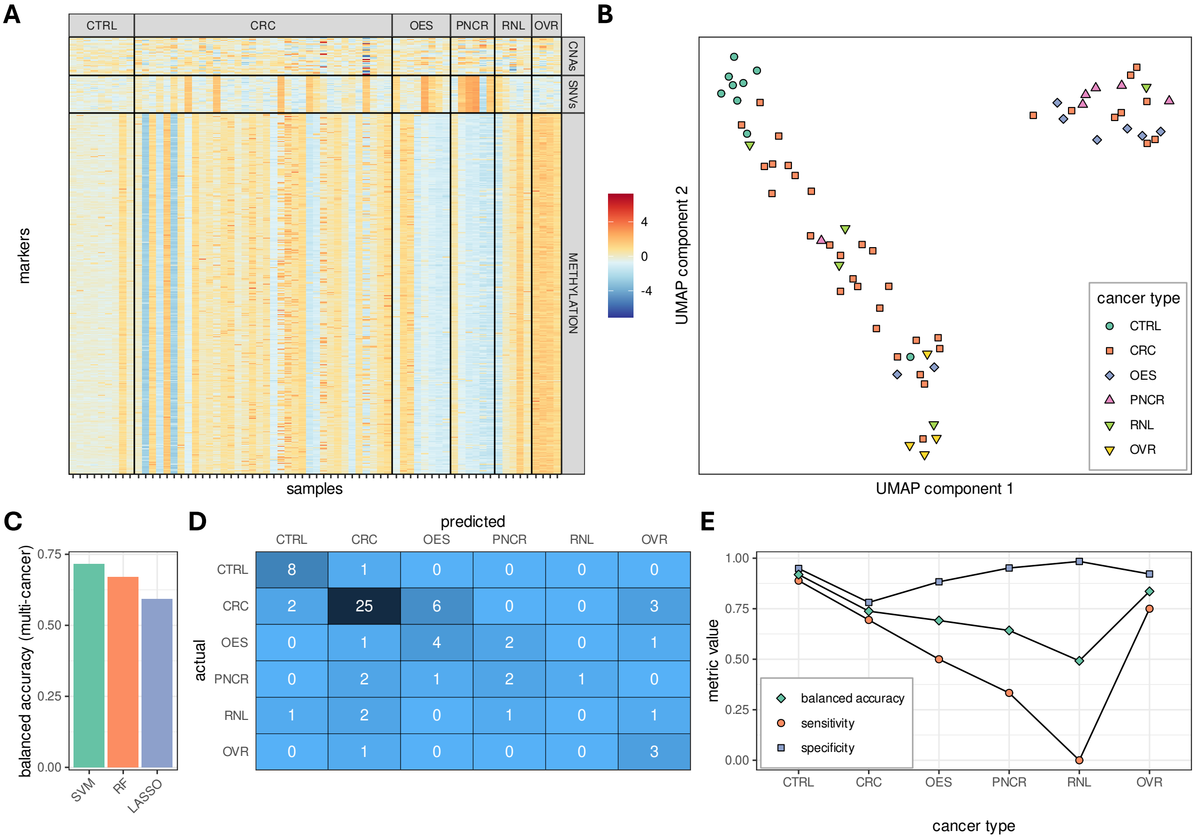

Supplementary Figure 6: *Prediction of cancer type/origin.* Chromosome arm-specific coverage (CNAs), chromosome arm-specific somatic single nucleotide variants (SNVs) and indels, and TCGA region-specific methylation signals for each sample were concatenated and used as predictors in three multi-cancer classifiers: Support Vector Machine (**SVM**), Random Forrest (**RF**) and Penalised Multinomial Regression (**LASSO**). A) The resulting data matrix is visually represented as a heatmap. B) Uniform Manifold Approximation and Projection (UMAP) of the concatenated data on the Cartesian plane. C) The performance (measured as the class-weighted balanced accuracy) of each classifier was assessed using Leave-One-Out Cross-Validation (**LOO-CV**). The best performing classifier was SVM, followed by RF and LASSO, with accuracies 71.7%, 65.6% and 59.3%, respectively. D) Confusion matrix, and E) sensitivity, specificity and balanced accuracy per sample group estimated using LOO-CV and the SVM classifier. **CTRL:** CBS controls (n = 9 subjects); **CRC:** colorectal (n = 36 subjects); **OES:** oesophageal (n = 8 subjects); **PNCR:** pancreatic (n = 6 subjects); **RNL:** renal (n = 5 subjects); **OVR:** ovarian (n = 2 subjects). Source data is provided as a source data file.

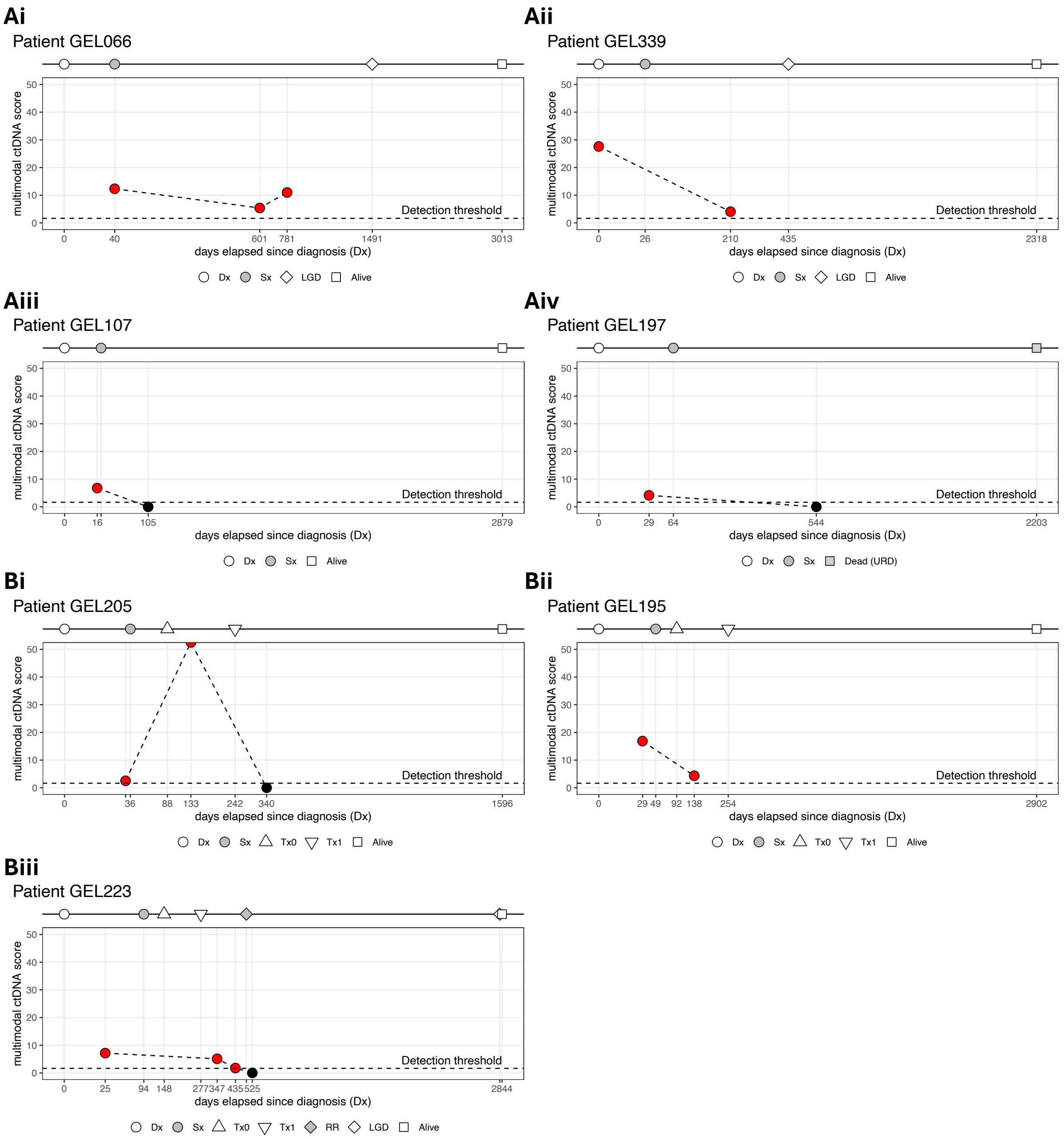

Supplementary Figure 7: *Multimodal ctDNA detection for post-operative MRD and adjuvant therapy response tracking in colorectal cancer without matched tumour.* A) Surgical patients who did not receive adjuvant treatment. Detection of ctDNA at the last post-surgery plasma sample correlated with the presence of pre-cancerous adenomas (Ai, Aii). Absence of detection of ctDNA at the last post-surgery plasma sample correlated with progression-free survival (Aiii, Aiv). Notice that case GEL197 died suddenly from unrelated reasons, 5 years after the last blood sample was collected. B) Surgical patients who received adjuvant treatment. In case GEL205 (Bi), a relatively high transient ctDNA burden was detected during adjuvant treatment, but there was no detectable ctDNA shortly after the last cycle of treatment and the patient was alive 4 years later. For case GEL195 (Bii), a post-operative blood sample collected 3 months after surgery indicated the presence of ctDNA in the plasma, although there was a 74% reduction in tumour burden compared to the pre-surgery sample (multimodal ctDNA score before surgery: 16.86,

after surgery: 4.34). Since the sample was taken 1½ months after the first cycle and approximately 4 months before the last cycle of adjuvant treatment, we presume that there would be no detectable ctDNA in the plasma after the end of therapy. Case GEL223 (Biii) did not show detectable ctDNA levels 8 months after the last cycle of adjuvant therapy, despite the diagnosis of prostate adenocarcinoma 1 month earlier. This was treated with hormone therapy and radiotherapy, and the patient was still alive 6½ years after the last plasma sample was collected. **Dx:** diagnosis; **Sx:** surgery; **Tx0:** first cycle of adjuvant therapy; **Tx1:** last cycle of adjuvant therapy; **RR:** recurrence; **LGD:** low-grade dysplasia; **URD:** unrelated death. Source data is provided as a source data file.

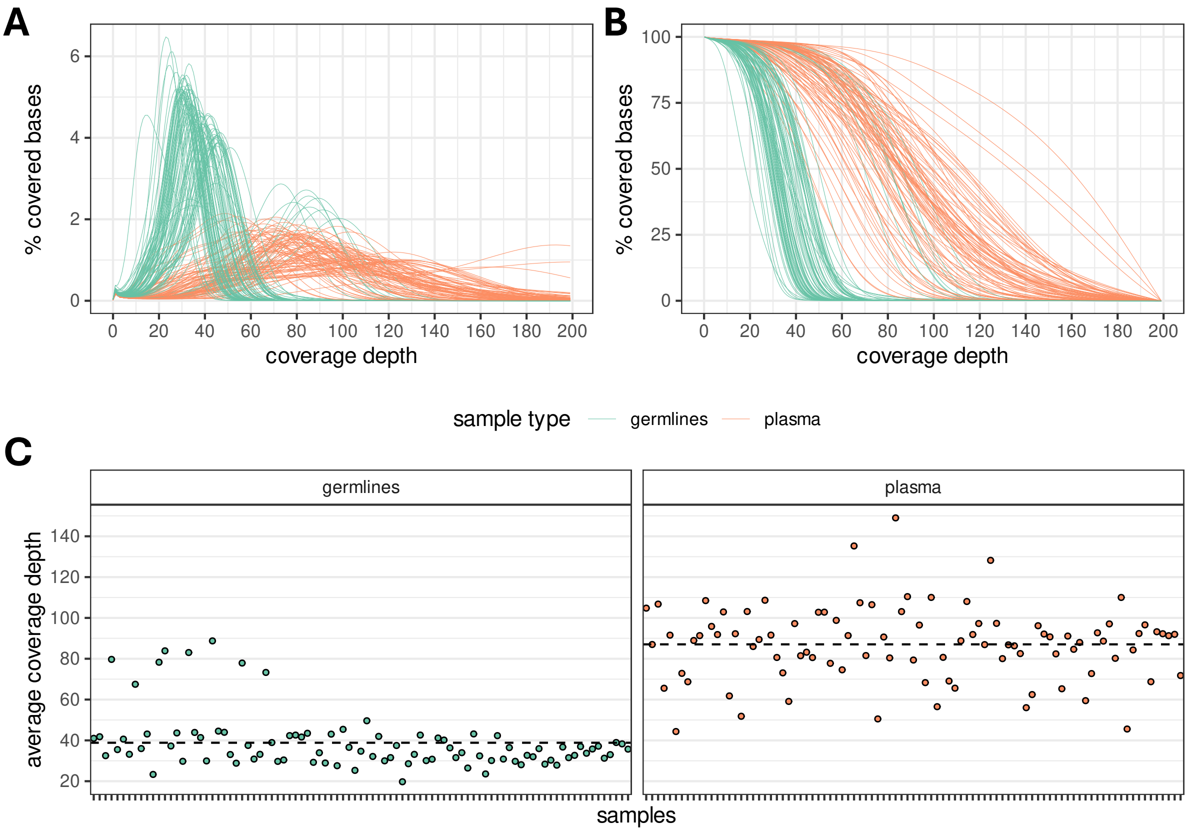

Supplementary Figure 8: ***Overview of the depth of coverage of the sequencing data.*** A) Distribution and B) one minus the cumulative distribution of the genome-wide coverage in germline and baseline plasma samples. C) Average coverage depth per sample in germline and plasma samples. Target coverage was 30x and 80x in germline (n = 91) and plasma (n = 91) samples, respectively. Source data is provided as a source data file.
