# Supplementary material for: Multimodal cell-free DNA whole-genome TAPS is sensitive and reveals specific cancer signals
Source: Nat Commun. 2025 Jan 8;16:430. doi: 10.1038/s41467-024-55428-y (PMC11711490; doi:10.1038/s41467-024-55428-y)
Supplement: Supplementary file 3 — Reporting Summary [file 41467_2024_55428_MOESM3_ESM.pdf]

nature portfolio

Corresponding author(s): Prof. Anna Schuh
Dr. Dimitris Vavoulis

Last updated by author(s): 28/10/2024

Reporting Summary

Nature Portfolio wishes to improve the reproducibility of the work that we publish. This form provides structure for consistency and transparency in reporting. For further information on Nature Portfolio policies, see our Editorial Policies and the Editorial Policy Checklist.

Statistics

For all statistical analyses, confirm that the following items are present in the figure legend, table legend, main text, or Methods section.

n/a	Confirmed	
☐	☒	The exact sample size (*n*) for each experimental group/condition, given as a discrete number and unit of measurement
☐	☒	A statement on whether measurements were taken from distinct samples or whether the same sample was measured repeatedly
☐	☒	The statistical test(s) used AND whether they are one- or two-sided<br>*Only common tests should be described solely by name; describe more complex techniques in the Methods section.*
☐	☒	A description of all covariates tested
☐	☒	A description of any assumptions or corrections, such as tests of normality and adjustment for multiple comparisons
☐	☒	A full description of the statistical parameters including central tendency (e.g. means) or other basic estimates (e.g. regression coefficient) AND variation (e.g. standard deviation) or associated estimates of uncertainty (e.g. confidence intervals)
☐	☒	For null hypothesis testing, the test statistic (e.g. *F*, *t*, *r*) with confidence intervals, effect sizes, degrees of freedom and *P* value noted<br>*Give P values as exact values whenever suitable.*
☐	☒	For Bayesian analysis, information on the choice of priors and Markov chain Monte Carlo settings
☐	☒	For hierarchical and complex designs, identification of the appropriate level for tests and full reporting of outcomes
☐	☒	Estimates of effect sizes (e.g. Cohen's *d*, Pearson's *r*), indicating how they were calculated

Our web collection on statistics for biologists contains articles on many of the points above.

Software and code

Policy information about availability of computer code

Data collection	Not applicable
Data analysis	bwa mem v0.7.17<br>bedtools interect v2.30.0<br>bcftools mpileup v1.15<br>R v4.1.3<br>mgcv v1.8-40 (R package)<br>signal v0.7-7 (R package)<br>DNAcopy v1.68.0 (R package)<br>astair v3.3.2<br>randomForest v4.7.1<br>e1071 v1.7<br>glmnet v4.1-7

For manuscripts utilizing custom algorithms or software that are central to the research but not yet described in published literature, software must be made available to editors and reviewers. We strongly encourage code deposition in a community repository (e.g. GitHub). See the Nature Portfolio guidelines for submitting code & software for further information.

Data

Policy information about availability of data

All manuscripts must include a data availability statement. This statement should provide the following information, where applicable:

- Accession codes, unique identifiers, or web links for publicly available datasets
- A description of any restrictions on data availability
- For clinical datasets or third party data, please ensure that the statement adheres to our policy

Anonymised clinical and sequencing data used in this study have been deposited in the European Genome-Phenome Archive (EGA) under accession codes XXX, YYY and ZZZ. See data availability statement in the manuscript for more details.

Research involving human participants, their data, or biological material

Policy information about studies with human participants or human data. See also policy information about sex, gender (identity/presentation), and sexual orientation and race, ethnicity and racism.

Reporting on sex and gender	We included cancer samples from biological males (n=38) and females (n=23). Sex was self-reported. We included, where available, breast and ovarian cancer samples from biological females, thus results from the analysis of these samples are restricted to this particular sex. We did not include prostate cancer as samples were not available.
Reporting on race, ethnicity, or other socially relevant groupings	Samples were collected via the Oxford University Hospital NHS trust as part of the Genomics England 100,000 genome program and as part of the SCAN study, both of which were conducted in England's National Health Service. Overall, 13% of patients recruited into this program were from non-white ethnic groups. This is similar to the percentage of people identifying as non-white in the general population in England. Patients recruited in this study identified exclusively as White British, which is consistent with Oxford's demographics.
Population characteristics	The group of patients recruited in this study was relatively homogeneous in terms of their demographic characteristics. All patients identified exclusively as White British, which is consistent with Oxford's demographics, and median age was 68 years old. Both biological sexes were represented in comparable numbers (see above). In each cancer type, cancer stage was the only confounding variable, with both early- and late-stage cancer patients recruited in comparable numbers (n=25 vs n=36, respectively).
Recruitment	All patients presenting with symptoms of cancer via rapid diagnostics clinics or the oncology clinics at Oxford University Hospital NHS trust were approached to participate in this study. Samples were stored and patients were followed up via hospital records for 5 years or more. Patients subsequently diagnosed with cancer were selected at random making sure that both early- and late-cancer stages were represented in our cohort in numbers comparable to those of the most common cancer types in the population. Age- and sex-matched subjects without cancer were also selected at random for this case-control study.
Ethics oversight	The National Health Service (NHS) Health Research Authority South Central—Oxford C Research Ethics Committee approved this study, and all research was performed in accordance with relevant regulations and guidelines and with the Declaration of Helsinki. Written informed consent was obtained for patients recruited into the 100,000 Genomics England (GEL) pilot study and the rapid diagnostic clinic research pathway in Oxford called Suspected CANcer (SCAN) according to Oxford Radcliffe Biobank (ORB) guidelines (Oxford C Research Ethics Committee Number: 19/SC/0173). For 9 additional non-cancer controls, blood was received from Cambridge Bioscience Human Blood Products Supply Service, where comprehensive informed 359 written consent was provided 360 in accordance with UK ethics and consent regulations.

Note that full information on the approval of the study protocol must also be provided in the manuscript.

Field-specific reporting

Please select the one below that is the best fit for your research. If you are not sure, read the appropriate sections before making your selection.

☒ Life sciences ☐ Behavioural & social sciences ☐ Ecological, evolutionary & environmental sciences

For a reference copy of the document with all sections, see nature.com/documents/nr-reporting-summary-flat.pdf

Life sciences study design

All studies must disclose on these points even when the disclosure is negative.

Sample size	We used samples from 61 cancer and 30 non-cancer subjects resulting in a total of 214 genomes sequenced at 30x (germlines) or at least 80x (liquid biopsies). Sample size was determined by sample availability and budget considerations
Data exclusions	No data were excluded

Replication	Not applicable. Samples were collected from different patients resulting in a total of 61 cancer and 30 non-cancer biological replicates. In addition, technical replicates were not needed as the sequencing noise and artifacts were controlled for through bioinformatics approaches
Randomization	Not applicable. This is a non-interventional, descriptive case-control study. Therefore, randomization is not required.
Blinding	Not applicable. This is a non-interventional, descriptive case-control study. Therefore, blinding is not required.

Reporting for specific materials, systems and methods

We require information from authors about some types of materials, experimental systems and methods used in many studies. Here, indicate whether each material, system or method listed is relevant to your study. If you are not sure if a list item applies to your research, read the appropriate section before selecting a response.

Materials & experimental systems

n/a	Involved in the study	
☒	☐	Antibodies
☒	☐	Eukaryotic cell lines
☒	☐	Palaeontology and archaeology
☒	☐	Animals and other organisms
☐	☒	Clinical data
☒	☐	Dual use research of concern
☒	☐	Plants

Methods

n/a	Involved in the study	
☒	☐	ChIP-seq
☒	☐	Flow cytometry
☒	☐	MRI-based neuroimaging

Clinical data

Policy information about clinical studies

All manuscripts should comply with the ICMJE guidelines for publication of clinical research and a completed CONSORT checklist must be included with all submissions.

Clinical trial registration	Clinical studies that are of relevance to this paper are:<br><br>1) The Genomics England (GEL) Cancer Pilot (https://www.genomicsengland.co.uk/initiatives/100000-genomes-project)<br>2) The Suspected CANcer (SCAN) Pathway (http://doi.org/10.1136/bmjopen-2017-018168)<br><br>Neither of the above is an interventional clinical trial and for this reason there is no associated clinical trial registration
Study protocol	1) For the GEL Cancer Pilot, see https://doi.org/10.1038/gim.2017.241<br>2) For the SCAN pathway, see http://doi.org/10.1136/bmjopen-2017-018168
Data collection	see Recruitment section above
Outcomes	The main outcomes of relevance to this paper were cancer type and cancer stage. Cancer type was determined through the NHS pathology department. Cancer stages were determined according to RECIST TNM criteria.

Plants

Seed stocks	N/A
Novel plant genotypes	N/A
Authentication	N/A
