# Supplementary material for: Multimodal cell-free DNA whole-genome TAPS is sensitive and reveals specific cancer signals
Source: Nat Commun. 2025 Jan 8;16:430. doi: 10.1038/s41467-024-55428-y (PMC11711490; doi:10.1038/s41467-024-55428-y)
Supplement: Supplementary file 4 — Description of Additional Supplementary Files [file 41467_2024_55428_MOESM4_ESM.pdf]

Description of Additional Supplementary Files

Supplementary Data 1

Description: Clinical characteristics of the 61 cancer patients used in this study. Subjects from this cohort were symptomatic patients referred for urgent investigation for a possible gynaecological, lower, or upper GI or renal cancer, or to a rapid diagnostic centre (RDC) with non-specific symptoms, which were later diagnosed with cancer, and referred to surgery with curative intent.

Supplementary Data 2

Description: 2113 hyper-methylated regions from 5 TCGA studies (COAD, ESCA, KIRC, KIRP, PAAD). 377 among them were kept in the final analysis, after denoising. Genomic coordinates are 0-based.
